# Is Colchicine a New Game-Changer in Patients With Acute Coronary Syndrome?

**DOI:** 10.7759/cureus.22874

**Published:** 2022-03-05

**Authors:** Abdul Q Nawabi, Warda Hassan, Lijuan Chen, Naveed Shaikh, Kiran Abbas, Fatima T Zehra

**Affiliations:** 1 Cardiology, Zhongda Hospital Southeast University, Nanjing, CHN; 2 Internal Medicine, Dow University of Health Sciences, Karachi, PAK; 3 Cardiology, National Institute of Cardiovascular Diseases, Karachi, PAK; 4 Internal Medicine, Jinnah Postgraduate Medical Centre, Karachi, PAK; 5 Internal Medicine, Jinnah Sindh Medical University, Karachi, PAK

**Keywords:** cardiovascular diseases, acute coronary syndrome, colchicine, vascular inflammation, myocardial infarction

## Abstract

Colchicine, an anti-inflammatory drug, was declared as a potently cheap and effective drug in treating atherosclerosis. This report is a detailed understanding and an in-depth interpretation of the colchicine cardiovascular outcomes trial (COLCOT) that has taken place in recent years. It is a secondary quantitative study that has reviewed studies discussing the role of colchicine in myocardial infarction, inflammation, ST-elevation myocardial infarction, and in general, acute coronary syndrome (ACS). Different trials statistically proved colchicine's role in ACS by lowering the levels of high c-reactive protein, decreasing low attenuation plaque volume, and stabilizing plaque. Hence, in other words, it has been revealed that this drug has potential in the management of ACS. Colchicine reported promising results in reducing the risk of recurrent myocardial infarction, stroke, and sudden cardiac arrest, playing a massive role in lowering inflammation and the mortality caused by cardiovascular diseases.

## Introduction and background

Inflammation is one of the main contributing factors to cardiovascular system (CVS) disorders. Colchicine which is extracted from a plant known as a "meadow saffron" was historically used for the treatment of swelling [1]. Moreover, its extract has been used for the healing of gout by pharmacologists and physicians in Greece 2000 years ago. It is a cheap and effective drug, well-known to treat a vast array of diseases [2].

There have been many studies highlighting the role of colchicine in cardiac diseases mainly as an anti-inflammatory drug. For instance, in 1987 at the Sant Pau Hospital, Barcelona, it was reported that colchicine helped in the healing of recurrent pericarditis [2]. The benefits of colchicine for the treatment of CVS diseases were evaluated in 1992 by O’Keefe in a small trial where its efficacy was observed in preventing restenosis after elective angioplasty [3]. The authors reported that colchicine did not benefit in preventing restenosis after elective angioplasty. However, later in 2013, two randomized clinical trials (RCTs) reported contradictory findings claiming that using colchicine in steady low-doses provides significant benefit to the patients with stable coronary artery disease as well as in patients with diabetes undergoing angioplasty [2,4].

Inflammation plays a substantial role in all stages of the development and progression of atherosclerotic plaque by promoting plaque instability leading to acute coronary syndrome (ACS). In coronary arteries, the inflammation is not just limited to the main culprit lesion, but consistent inflammatory cell activation has been found at locations that are further away from the main plaque rupture. This may contribute to recurrent plaque rupture and recurrent cardiovascular events, even if the patient is given optimal medical therapy [1,2]. Therefore, despite adequate pharmacological therapy, patients remain at high risk of developing a subsequent cardiovascular event. Recurrent events in these patients are particularly troublesome because they are correlated with considerably higher morbidity and mortality compared to the primary event.

Statins are regarded as optimal medical therapy for the treatment and management of patients with ACS. Apart from their lipid-lowering properties, statins are well-known as an anti-inflammatory drug in action. Nevertheless, scientists are focusing more on developing pharmacological therapies that explicitly target inflammation which will further enhance outcomes in high-risk patients [5,6]. Later, the Canakinumab Anti-inflammatory Thrombosis Outcome Study (CANTOS) reported that a steady dose of canakinumab, an anti-inflammatory drug, reduced major adverse cardiovascular events (MACEs) among patients with a history of myocardial infarction (MI) and lowered the levels of c-reactive proteins (CRP) [7]. However, the use of canakinumab was limited because of the higher rate of fatal infections and the high cost. Therefore, there was a critical necessity for new remedies in patients with a history of MI. Colchicine is a commonly used and very effective anti-inflammatory drug; its low dose has been proven to be efficacious, safe, and well-tolerated by patients of the ACS among other cardiovascular disorders [3,4,8].

Many studies also illustrated the association that had been made between thrombus formation and plaque rupture, these were cases that were found in acute MI and in individuals who died because of non-cardiac causes [9-11]. The atherosclerotic plaques are composed of inflammatory cells with a central lipid core. If a plaque is found in coronary arteries, it is regarded as “coronary atheromata” which is often a result of the weakening of collagen structures and is hammered and shaped by the inflammatory processes and mechanisms that take place within the plaque [11].

Atherothrombosis, secondary to inflammation, is accompanied by thrombus formation and plaque disruption which can lead to cardiovascular death. Over the years, studies were focused on trying to determine whether lowering inflammation leads to reducing the risk of plaque rupture or not, however, none of the studies were able to prove that reducing the inflammation would also lessen the risk and deaths of cardiovascular rates. Colchicine plays a vital role in these patients as it is an inexpensive, well-tolerated medication that mainly prevents microtubule polymerization in turn inhibiting or reducing the inflammatory processes [12].

This drug was initially used as intrapericardial injections in 2007. Later, oral pills of colchicine were also introduced. Hence, when it comes to oral dosages, the oral preparation that is used in the United States of America is 0.6 mg of colchicine although in other countries 0.5 mg is also used, therefore it can be denoted from this fact that in every country, every type of heart disease, the disease, and the therapy would differ [13].

It is evident through several studies that colchicine is useful in a wide range of diverse spectrum of CVS diseases. The Colchicine in Acute Pericarditis (COPE) and Colchicine for Recurrent Pericarditis (CORE) studies have highlighted the benefits of using colchicine as adjunctive therapy for the treatment of consistent pericarditis, where colchicine was used as an adjunct therapy to non-steroidal anti-inflammatory drugs (NSAIDs), proving to be a very useful and effective addition to the clinical setting for this treatment. In the COPE trial, colchicine was an addition to the aspirin therapy given to the 120 patients who had the first episode of acute pericarditis, and it was associated with fast relief from the symptoms of this consistent pain. There were 36.7% of patients, who were in the aspirin group compared to 11.7% in the aspirin plus colchicine group (given to them after seven hours). It was observed that 32.3% of patients taking aspirin and colchicine experienced significantly reduced episodes of pericarditis compared to patients only taking aspirin. Colchicine had also been preferred over corticosteroids and NSAIDs [14].

Not many updated comprehensive studies are present that evaluated the efficacy of colchicine in the management of ACS. Therefore, considering the dearth of local and international data, in this review, we have critically discussed the role of colchicine in the long-term treatment of ACS and whether it reduces the mortality and complications of the ACS.

## Review

Acute coronary syndrome and inflammation

Widespread evidence supports that inflammation plays a significant role in the development and progression of ACS, and understanding the pathogenesis behind this phenomenon would help improve the patient outcome which may lead to lower mortality. Endothelial activation promotes the release of proinflammatory cytokines, oxygen-free radicals, and matrix-degrading metalloproteinases, which magnify the nearby inflammatory processes and aid in lowering the plaque stabilization via collagen-mediated deterioration of the fibrous cap [15,16]. The major contributor elements causing inflammation in ACS are the proinflammatory cytokines that play a critical role in endothelial activation, subsequently leading to inflammatory cell recruitment and activation of endothelial cells and neutrophils. Moreover, plaque rupture results in thrombosis [17].

The nucleotide-binding oligomerization domain (NOD)-like receptor protein 3 (NLRP3) inflammasome is a key part of the innate immune system. It is present in myeloid cells including the neutrophils. The inflammasome is activated in a two-phase process. First, the exposure to cholesterol crystals which are found in high amounts in an unstable plaque is detected by the NLRP3 receptor, which primes the inflammasome complex, subsequently causing the assembly of NLRP3, which contains the caspase recruitment domain, and the effector cysteine protease procaspase-1. Second, adenosine triphosphate leads to activation of caspase-1 and secretion of active IL-1β and IL-18 [18,19]. In short, exposure to stimuli, such as cholesterol crystals, which are found in abundant quantities in unstable plaque, triggers the NLRP3 inflammasome. This in turn releases the two key inflammatory cytokines (IL-1β and IL-18), both of which are associated with cardiovascular events and contribute significantly to mediating the development and progression of plaque destabilization.

Anti-inflammatory mechanism of colchicine

Colchicine is a lipid-soluble drug that is absorbed by the jejunum and ileum. It is a standard anti-mitotic drug that freezes mitotic cells in metaphase. It binds to soluble tubulin to form tubulin-colchicine complexes, which then bind to the ends of microtubules to prevent the elongation of the microtubule polymer. The mechanisms of colchicine can be summarized as in Table 1 [20].

**Table 1:** Summary of mechanisms of action of colchicine.

Mechanisms of colchicine
Inhibition of activation of innate immunity, NALP3 inflammasome activation, caspase-1 activation; inhibition of release of chemotactic factor from neutrophils resulting in neutrophil recruitment.
At low dilutions, colchicine aids in inhibiting the expression of E-selectin on endothelial cells preventing adhesion of neutrophils.
At high dilutions, colchicine aids in furthering the casting off L-selectin from neutrophils preventing additional recruitment.
Colchicine causes inhibition of neutrophil activation and release of IL1, IL8, and superoxide. Subsequently, promoting the growth of dendritic cells which act as antigen-presenting cells. Colchicine inhibits the proliferation of vascular endothelial growth factor (VEGF) hence, endothelial proliferation.

This drug not only helps in reducing inflammation but is also responsible for reducing leukocyte activation by suppressing NLRP3 inflammasome, leading to disruption in the formation of microtubules by impairing the mitochondrial localization of the inflammasome proteins resulting in cytokine production. The activation of interleukin-1β (IL-1β) and IL‐18 due to cellular stress plays a key role in the formation of plaque and causing destabilization [21]. This further leads to increased CRP which enhances cardiovascular deterioration. Hence, colchicine provides a cost-effective and easily available alternative to lessen the inflammation in any cardiovascular event thus preventing further damage. It does so by interfering with leukocyte activation as mentioned previously, in turn, causing cell breakdown via mitochondrial colocalization, resulting in cytokine production [22].

The colchicine cardiovascular outcome trial

Between December 2015 and August 2018, the Colchicine Cardiovascular Outcomes Trial (COLCOT) was conducted to determine the ability of colchicine to lower the cardiovascular events in patients with a recent MI compared to the placebo group [12]. This double-blinded RCT enrolled a total of 4745 patients with 2366 of them being appointed to the colchicine group and 2379 to the placebo group. The median follow-up was for 22.6 months. The colchicine group received a dose of 0.5 mg once daily and the control group received a placebo of similar consistency daily. All patients who were enrolled in the trial had an MI within 30 days of the trial and were treated according to the standard protocol including the use of statins [12].

The authors identified death from cardiovascular causes, resuscitation upon cardiac arrest, subsequent MI, stroke, or frequency of hospitalization for cardiac-related causes as the primary endpoints for the trial. The all-cause mortality was assessed for both groups. Additionally, the difference of CRP levels from baseline to six months and the difference of white blood cell count from baseline to 12 months were also recorded for the colchicine and the placebo groups - these two are the biomarkers of inflammation [12].

Efficacy of colchicine in reducing major adverse cardiovascular events

The results of the COLCOT showed that the colchicine group experienced a significantly lower number of primary end-point events (5.5%) compared to 7.1% in the placebo group (p=0.02). The secondary outcomes revealed that there were 20 (0.8%) deaths due to cardiovascular causes in the colchicine group compared to the 24 (1.0%) patients who died from the placebo group. The incidence of stroke in the colchicine group was 0.2% compared to 0.8% in the placebo group. Similarly, the need for urgent hospitalization for revascularization was 1.1% in the colchicine group compared to a higher frequency of 2.1% in the placebo group. The results of the COLCOT are backed up by a previous promising trial, conducted in 2013, where the author evaluated the efficacy of low dose colchicine in over five hundred patients with stable CAD. It was revealed that colchicine administered in low doses in addition to statin therapy and optimal medical therapy (OMT) was successful in the deterrence of any major cardiovascular event (hazard ratio=0.33, p<0.001) [4].

An observational study was carried out to assess the impact of colchicine on ACS, this study consisted of 80 patients who were given a dose of 0.5 mg every day for around a month. The computed tomography coronary angiography was performed at baseline for this one year. This study also included OMT and they were being given statin therapy which had a level of <1.8 mmol/L. They wanted to observe a change in LAP (which is a low attenuation plaque), the flexibility of high sensitivity c-reactive protein (hsCRP), and change atheroma volume. LAP has a very powerful and successful future for imaging studies which are good for ACS [23]. In a study, colchicine was proven to be fruitful in patients with unhealthy lifestyles including patients who were smokers, diabetics, and even underweight. Colchicine therapy proved to be effective for all these individuals with a minority having adverse effects [24].

Role of colchicine in reducing inflammation in ACS

In a 2007 study by Nidorf and Thompson, it was revealed that low dose colchicine effectively and autonomously lowered the levels of hsCRP levels in patients with stable coronary artery disease. Hs-CRP is a biomarker of inflammation in patients with cardiovascular disease [25]. As stated earlier, the COLCOT assessed the difference in hsCRP levels and white blood cell counts in both the colchicine group and the placebo group. However, due to a small number of patients, i.e., 207, the interpretation of these results is limited. Similarly, the data about the change in white blood cell counts in both groups were also available for a relatively small subgroup of 1972 patients. Therefore, the findings should be cautiously interpreted.

However, a recent similar study conducted in 2018 showed that low doses of colchicine (0.5 mg per day) for twelve months in conjunction with optimum medical therapy caused a significant reduction in LAP volume in the colchicine group (p=0.008) and also in hsCRP levels (p<0.0001) [23].

These findings altogether indicate that steady doses of colchicine do play a part in lowering the levels of CRP in patients with a history of MI and aids in the stabilization of plaque. A review conducted by Vaidya concluded that colchicine recently gained a footing in cardiovascular medicine and may have the potential to reduce the inflammation resulting in plaque stabilization that translates into slowing down the progression of atherothrombosis, thereby reducing the rate of recurrent cardiovascular events and improving patient outcomes [26].

Adverse effects of colchicine

Colchicine, a readily available and inexpensive drug, safely employs its potent anti-inflammatory effects in targeting inflammation. The COLCOT, with a statistically insignificant difference of 0.89, revealed that the 372 (16%) patients in the colchicine group and the 371 (15.8%) patients in the placebo group experienced few adverse events, indicating that colchicine is a safe drug. Hence, it can be administered in low doses of 0.5 mg per day to patients with a recent MI [12].

A trivial amount of 2.2% (n=51) of the patients who received colchicine suffered from infection compared to the 1.6% (n=38) of patients in the placebo group. However, the difference was statistically insignificant (p=0.15). Furthermore, as an aftereffect of the medication, it was reported that 43 patients (1.8%) of the colchicine group and 46 (2%) of the placebo group were also diagnosed with cancer, with an insignificant difference of p=0.77.

The frequency of patients who suffered from nausea and flatulence were both significantly higher in the colchicine group as compared to the placebo group with a p<0.05. Another serious adverse event was pneumonia, which was also reportedly higher among patients who were administered colchicine (n=21, 0.9%) in contrast to the nine patients (0.4%) of the placebo group with p=0.03. Another commonly experienced adverse effect was diarrhea (9.7% of the colchicine group compared to 8.9% of the placebo group) [25,26].

## Conclusions

In conclusion, it is quite evident that inflammation played a vital role in the pathogenesis of acute coronary syndrome (ACS) and atherosclerotic events, which was then targeted by multiple medications in the last few years. However, none of them proved to be beneficial in improving patient outcomes when investigated in largely centered trials. On the contrary, the advent of the recently conducted large-scale COLCOT study along with other new trials evaluating the efficacy of colchicine reported promising results that patients receiving colchicine had a reduced risk of recurrent MI, stroke, and sudden cardiac arrest. Hence, colchicine plays a massive role in lowering inflammation and the mortality caused by CVS diseases. Further longitudinal studies are required to study the long-term outcome of patients using colchicine.
